# Long-term outcomes of children with neonatal transfer: the Japan Environment and Children’s Study

**DOI:** 10.1007/s00431-022-04450-7

**Published:** 2022-03-25

**Authors:** Katsuya Hirata, Kimiko Ueda, Kazuko Wada, Satoyo Ikehara, Kanami Tanigawa, Tadashi Kimura, Keiichi Ozono, Hiroyasu Iso, the Japan Environment and Children’s Study Group, Michihiro Kamijima, Shin Yamazaki, Yukihiro Ohya, Reiko Kishi, Nobuo Yaegashi, Koichi Hashimoto, Chisato Mori, Shuichi Ito, Zentaro Yamagata, Hidekuni Inadera, Takeo Nakayama, Hiroyasu Iso, Masayuki Shima, Hiroshige Nakamura, Narufumi Suganuma, Koichi Kusuhara, Takahiko Katoh

**Affiliations:** 1 Department of Neonatal Medicine, Osaka Women’s and Children’s Hospital, 840 Murodo-cho, Izumi, Osaka 594-1101 Japan; 2 Osaka Maternal and Child Health Information Center, Osaka Women’s and Children’s Hospital, Izumi, Osaka Japan; 3 Public Health, Department of Social Medicine, Osaka University Graduate School of Medicine, Suita, Osaka Japan; 4 Department of Obstetrics and Gynecology, Osaka University Graduate School of Medicine, Suita, Osaka Japan; 5 Department of Pediatrics, Osaka University Graduate School of Medicine, Suita, Osaka Japan

**Keywords:** Inborn, Neonatal cardiopulmonary resuscitation, Neonatal transport, Outborn, Perinatal system

## Abstract

This study aimed to evaluate the association of neonatal transfer with the risk of neurodevelopmental outcomes at 3 years of age. Data were obtained from the Japan Environment and Children’s Study. A general population of 103,060 pregnancies with 104,062 fetuses was enrolled in the study in 15 Regional Centers between January 2011 and March 2014. Live-born singletons at various gestational ages, including term infants, without congenital anomalies who were followed up until 3 years were included. Neurodevelopmental impairment was assessed using the Ages and Stages Questionnaire, third edition (ASQ-3) at 3 years of age. Logistic regression was used to estimate the adjusted risk and 95% confidence interval (CI) for newborns with neonatal transfer. Socioeconomic and perinatal factors were included as potential confounders in the analysis. Among 83,855 live-born singletons without congenital anomalies, 65,710 children were studied. Among them, 2780 (4.2%) were transferred in the neonatal period. After adjustment for potential confounders, the incidence of neurodevelopmental impairment (scores below the cut-off value of all 5 domains in the ASQ-3) was higher in children with neonatal transfer compared with those without neonatal transfer (communication: 6.5% vs 3.5%, OR 1.42, 95% CI 1.19–1.70; gross motor: 7.6% vs 4.0%, OR 1.26, 95% CI 1.07–1.49; fine motor: 11.3% vs 7.1%, OR 1.19, 95% CI 1.03–1.36; problem solving: 10.8% vs 6.8%, OR 1.29, 95% CI 1.12–1.48; and personal-social: 6.2% vs 2.9%, OR 1.52, 95% CI 1.26–1.83).

*   Conclusion*: Neonatal transfer was associated with a higher risk of neurodevelopmental impairment at 3 years of age.

**Table:** 

**What is Known:** *• Neonatal transfer after birth in preterm infants is associated with adverse short-term outcomes.* *• Long-term outcomes of outborn infants with neonatal transfer in the general population remain unclear.*	
**What is New:** *• This study suggests that neonatal transfer at birth is associated with an increased risk of neurodevelopmental impairment.* • *Efforts for referring high-risk pregnant women to higher level centers may reduce the incidence of neonatal transfer, leading to improved neurological outcomes in the general population.*

**Supplementary information:**

The online version contains supplementary material available at 10.1007/s00431-022-04450-7.

## Introduction

Delivery of neonates is not always safe. Preterm neonates usually require resuscitation at birth and need to be admitted to neonatal intensive care units (NICUs). Even in term neonates, approximately 5% require positive pressure ventilation or intubation at birth [1, 2]. Morbidity and mortality differ in high-risk neonates with levels of NICUs where they were treated [3–7]. Centralization of high-risk delivery to hospitals with the highest level of neonatal care is optimal for better neonatal outcomes [8]. Traditionally, in Japan, approximately 50% of deliveries are managed at small birth centers or level I hospitals for maternal care where a pediatrician is not always available at delivery [1, 9]. This is quite different from North America, where a pediatrician attends almost all deliveries in general hospitals [1]. Transporting women with high-risk pregnancy or at risk of preterm births to tertiary centers are recommended. However, postnatal transport of sick neonates to higher-level institutes cannot be totally avoided because all risk cannot be anticipated prenatally.

Neonatal transfer after birth in preterm infants is associated with adverse short-term outcomes, especially for brain injury, such as intraventricular hemorrhage and periventricular leukomalacia [9–18]. Only limited reports have focused on long-term outcomes of children who required neonatal transfer in the preterm population [17, 18]. No studies have focused on long-term outcomes of outborn infants who require neonatal transfer in the general population, mainly comprising term infants.

This study aimed to evaluate the association of neonatal transfer with the risk of neurodevelopmental outcomes at 3 years of age. We hypothesized that after adjusting for perinatal and socioeconomic confounders, the incidence of long-term neurodevelopmental impairment during 3 years from birth is higher in children who require neonatal transfer than in children without neonatal transfer.

## Patients and methods

### Study setting and population

The data used in the study were obtained from the Japan Environment and Children’s Study (JECS). The JECS is an ongoing nationwide, multicenter, prospective birth cohort study funded by the Ministry of the Environment, Japan [19, 20]. A general population of 103,060 pregnancies with 104,062 fetuses was enrolled in the JECS in 15 Regional Centers, covering a wide geographical area in Japan, between January 2011 and March 2014. Follow-up is planned until the children are 13 years of age to measure the effect of environmental factors on children’s health [19]. The detailed methodology has been previously reported [19, 20]. The JECS protocol was reviewed and approved by the Institutional Review Board on Epidemiological Studies of the Ministry of the Environment and the Ethics Committees of all participating institutions. Written informed consent was obtained from all participants. The present study was based on the dataset jecs-ta-20190930. Data used in the present study were perinatal data at birth and at 1 month of age. These data were transcribed from medical records by physicians, midwives/nurses, and/or Research Co-ordinators. Questionnaires were administered to enrolled mothers and their partners at first trimester and second/third trimester in pregnancy, and a month after birth [19]. Thereafter, questionnaires were sent out every 6 months [19]. Data used in the present study were obtained from questionnaires provided during pregnancy and at 3 years of age.

### Inclusion and exclusion criteria

Among 104,062 fetuses, live-born singletons were included in the study. Children with chromosomal anomalies or major congenital anomalies detected at birth or at 1 month of age were excluded [21]. Children who were followed up at 3 years and completed the 36-month Ages and Stages Questionnaire, third edition (ASQ-3) within the appropriate period (34.5–38 months) were included.

### Outcomes, exposures, and covariates

The primary outcome of the study was neurodevelopment as assessed using the ASQ-3 at 3 years. Each questionnaire contained 30 items divided into 5 developmental domains (6 items per domain) as follows: communication, gross motor skills, fine motor skills, problem solving, and personal-social [22]. The cutoff scores (− 2.0 SD) for each domain at 3 years of age were as follows: communication: 29.95, gross motor: 39.26, fine motor: 27.91, problem-solving: 30.03, personal-social: 29.89; these cutoff scores were based on previously validated cutoff scores for Japanese children [22]. Secondary outcomes were a physician’s diagnosis of neurodevelopmental delay, motor developmental delay, cerebral palsy, autism spectrum disorders, and epilepsy in children. These outcomes were collected from the questionnaire answered by caregivers when children were 3 years of age.

The exposure of interest was neonatal transfer, which was defined as inter-facility transport of the neonate. The following maternal variables were considered potential confounders: age, marital status, primigravida, fertility treatment, cesarean delivery, epidural analgesia during labor, hypertensive disorder of pregnancy (HDP), gestational diabetes mellitus (GDM), placenta previa, premature rupture of the membranes (PROM), intrauterine infection, intrauterine growth restriction (IUGR), non-reassuring fetal status (NRFS), alcohol drinking during pregnancy, smoking during pregnancy, educational status, work status, and household income. The following neonatal variables were considered potential confounders: gestational age (GA), birthweight, sex, and asphyxia at birth (5-min Apgar score < 7).

### Statistical analysis

Correlations between neonatal transfer and 3-year outcomes were assessed. Logistic regression was used to estimate the adjusted risk and 95% confidence interval (CI) for newborns with neonatal transfer. All of the factors shown in Tables 1 and 2 were included as potential confounders in the analysis. Missing values of some covariates (marital status, primigravida, fertility treatment, cesarean delivery, epidural analgesia during labor, asphyxia at birth, mother’s alcohol drinking during pregnancy, mother’s smoking during pregnancy, mother’s educational status, mother’s work status, and household income) were also included in the model by creating categories of such data using dummy variables to prevent a decrease in number. We assessed multicollinearity using variance inflation factors for each of the covariates. We considered that a variance inflation factor > 10 indicated potential multicollinearity. To exclude the effect of prematurity on the outcomes of neurological phenotypes, we conducted sensitivity analysis with cohorts stratified by GA. The full cohort was defined as a cohort including all children. The term cohort was defined as a cohort with a GA ≥ 37 weeks, and the preterm cohort was defined as a cohort with a GA < 37 weeks. We conducted a repeated analysis when we restricted the children with prolonged hospital stay (≥ 7 days) and asphyxia (5-min Apgar score < 7) at birth. Statistical significance was defined as *P* < 0.05. All statistical analyses were performed with EZR (Saitama Medical Center, Jichi Medical University, Saitama, Japan) [23], which is a graphical user interface for R software (The R Foundation for Statistical Computing, Vienna, Austria, version 4.1.0).

**Table 1:** Socioeconomic background characteristics in the full, term, and preterm cohorts with or without neonatal transfer

	Full cohort (all GAs)		Term cohort (GA ≥ 37 weeks)		Preterm cohort (GA < 37 weeks)	
	With neonatal transfer (*n* = 2780)	Without neonatal transfer (*n* = 62,930)	With neonatal transfer (*n* = 1799)	Without neonatal transfer (*n* = 61,259)	With neonatal transfer (*n* = 981)	Without neonatal transfer (*n* = 1671)
Maternal age*	32.2 (5.3)	31.4 (4.9)	32.1 (5.4)	31.4 (4.9)	32.4 (5.1)	31.8 (5.1)
Marital status: married						
Yes	2587 (93.1)	58,028 (92.2)	1679 (93.3)	56,494 (92.2)	908 (92.6)	1534 (91.8)
No	99 (3.6)	2595 (4.1)	55 (3.1)	2525 (4.1)	44 (4.5)	70 (4.2)
Missing data	94 (3.4)	2307 (3.7)	65 (3.6)	2240 (3.7)	29 (3.0)	67 (4.0)
Mother’s educational background						
Non-tertiary education	915 (32.9)	21,350 (33.9)	569 (31.6)	20,748 (33.9)	346 (35.3)	602 (36.0)
Tertiary education	1805 (64.9)	41,022 (65.2)	1216 (67.6)	39,975 (65.3)	589 (60.0)	1047 (62.7)
Missing data	60 (2.2)	558 (0.9)	14 (0.8)	536 (0.9)	46 (4.7)	22 (1.3)
Mother’s working status						
Working	1690 (60.8)	39,318 (62.5)	1094 (60.8)	38,268 (62.5)	596 (60.8)	1050 (62.8)
Student	13 (0.5)	344 (0.5)	9 (0.5)	336 (0.5)	4 (0.4)	8 (0.5)
Not working	929 (33.4)	19,542 (31.1)	600 (33.4)	19,047 (31.1)	329 (33.5)	495 (29.6)
Missing data	148 (5.3)	3726 (5.9)	96 (5.3)	3608 (5.9)	52 (5.3)	118 (7.1)
Household income						
< 4 million Japanese yen	965 (34.7)	22,957 (36.5)	601 (33.4)	22,313 (36.4)	364 (37.1)	644 (38.5)
≥ 4 and < 8 million Japanese yen	1282 (46.1)	29,167 (46.3)	870 (48.4)	28,428 (46.4)	412 (42.0)	739 (44.2)
≥ 8 million Japanese yen	298 (10.7)	6450 (10.2)	201 (11.2)	6296 (10.3)	97 (9.9)	154 (9.2)
Missing data	235 (8.5)	4356 (6.9)	127 (7.1)	4222 (6.9)	108 (11.0)	134 (8.0)
Mother’s drinking during pregnancy						
Yes	62 (2.2)	1688 (2.7)	42 (2.3)	1651 (2.7)	20 (2.0)	37 (2.2)
No	2649 (95.3)	60,571 (96.3)	1736 (96.5)	58,960 (96.2)	913 (93.1)	1611 (96.4)
Missing data	69 (2.5)	671 (1.1)	21 (1.2)	648 (1.1)	48 (4.9)	23 (1.4)
Mother’s smoking during pregnancy						
Yes	109 (3.9)	2831 (4.5)	73 (4.1)	2759 (4.5)	36 (3.7)	72 (4.3)
No	2595 (93.3)	58,232 (92.5)	1675 (93.1)	56,688 (92.5)	920 (93.8)	1544 (92.4)
Missing data	76 (2.7)	1867 (3.0)	51 (2.8)	1812 (3.0)	25 (2.5)	55 (3.3)

Data are expressed as mean (SD) or number (%)

*GA* gestational age

^*^Without missing data

**Table 2:** Perinatal characteristics of mothers and children in the full, term, and preterm cohorts with or without neonatal transfer

	Full cohort (all GAs)		Term cohort (GA ≥ 37 weeks)		Preterm cohort (GA < 37 weeks)	
	With neonatal transfer (*n* = 2780)	Without neonatal transfer (*n* = 62,930)	With neonatal transfer (*n* = 1799)	Without neonatal transfer (*n* = 61,259)	With neonatal transfer (*n* = 981)	Without neonatal transfer (*n* = 1671)
**Mothers**						
Primigravida						
Yes	1076 (38.7)	19,844 (31.5)	755 (42.0)	19,334 (31.6)	321 (32.7)	510 (30.5)
No	1665 (59.9)	42,407 (67.4)	1018 (56.6)	41,266 (67.4)	647 (66.0)	1141 (68.3)
Missing data	39 (1.4)	679 (1.1)	26 (1.4)	659 (1.1)	13 (1.3)	20 (1.2)
Fertility treatment						
Yes	240 (8.6)	5704 (9.1)	144 (8.0)	5563 (9.1)	96 (9.8)	141 (8.4)
No	2439 (87.7)	54,889 (87.2)	1588 (88.3)	53,426 (87.2)	851 (86.7)	1463 (87.6)
Missing data	101 (3.6)	2337 (3.7)	67 (3.7)	2270 (3.7)	34 (3.5)	67 (4.0)
Cesarean delivery						
Yes	1131 (40.7)	10,641 (16.9)	578 (32.1)	10,109 (16.5)	553 (56.4)	532 (31.8)
No	1646 (59.2)	52,183 (82.9)	1219 (67.8)	51,047 (83.3)	427 (43.5)	1136 (68.0)
Missing data	3 (0.1)	106 (0.2)	2 (0.1)	103 (0.2)	1 (0.1)	3 (0.2)
Epidural analgesia during labor						
Yes	55 (2)	1427 (2.3)	40 (2.2)	1395 (2.3)	15 (1.5)	32 (1.9)
No	2633 (94.7)	60,610 (96.3)	1719 (95.6)	59,012 (96.3)	914 (93.2)	1598 (95.6)
Missing data	92 (3.3)	893 (1.4)	40 (2.2)	852 (1.4)	52 (5.3)	41 (2.5)
Pregnancy-associated complications*						
Hypertensive disorder of pregnancy	295 (10.6)	1870 (3.0)	125 (6.9)	1693 (2.8)	170 (17.3)	177 (10.6)
Gestational diabetes mellitus	190 (6.8)	1481 (2.4)	148 (8.2)	1426 (2.3)	42 (4.3)	55 (3.3)
Placenta previa	89 (3.2)	289 (0.5)	29 (1.6)	242 (0.4)	60 (6.1)	47 (2.8)
Premature rupture of the membranes	474 (17.1)	4873 (7.7)	186 (10.3)	4494 (7.3)	288 (29.4)	379 (22.7)
Intrauterine infection	57 (2.1)	276 (0.4)	28 (1.6)	258 (0.4)	29 (3.0)	18 (1.1)
Intrauterine growth restriction	215 (7.7)	926 (1.5)	119 (6.6)	872 (1.4)	96 (9.8)	54 (3.2)
Non-reassuring fetal status	181 (6.5)	1349 (2.1)	107(5.9)	1291 (2.1)	74 (7.5)	58 (3.5)
**Children**						
Gestational age, weeks*	37.3 (3.2)	39.4 (1.2)	39.2 (1.3)	39.5 (1.1)	33.9 (2.8)	36.0 (1.2)
Term birth (≥ 37 weeks)	1798 (64.7)	61,247 (97.3)	1798 (100)	61,247 (100)	-	-
Late preterm birth (34, 35, 36 weeks)	624 (22.4)	1598 (2.5)	-	-	624 (63.6)	1598 (95.6)
Moderate preterm birth (32, 33 weeks)	171 (6.2)	38 (0)	-	-	171 (17.4)	38 (2.3)
Very preterm birth (< 32 weeks)	186 (6.7)	35 (0)	-	-	186 (19.0)	35 (2.1)
Birthweight, g*	2615 (679)	3052 (370)	2944 (487)	3068 (359)	2012 (558)	2503 (381)
Low birthweight (< 2500 g)	1146 (41.2)	3634 (5.8)	354 (19.7)	2852 (4.7)	792 (80.7)	782 (46.8)
Not low birthweight (≥ 2500 g)	1634 (58.8)	59,296 (94.2)	1445 (80.3)	58,407 (95.3)	189 (19.3)	889 (53.2)
Male sex*	1522 (54.7)	31,912 (50.7)	950 (52.8)	30,950 (50.5)	572 (58.3)	709 (57.6)
Apgar score, 5-min (< 7)						
Yes	132 (4.7)	141 (0.2)	62 (3.4)	123 (0.2)	70 (7.1)	18 (1.1)
No	2601 (93.6)	60,259 (96.2)	1704 (94.7)	58,952 (96.2)	897 (91.4)	1577 (94.4)
Missing data	47 (1.7)	2260 (3.6)	33 (1.8)	2184 (3.6)	14 (1.4)	76 (4.5)

Data are expressed as mean (SD) or number (%)

*GA* gestational age

^*^Without missing data

## Results

### Population characteristics

**Fig. 1:**
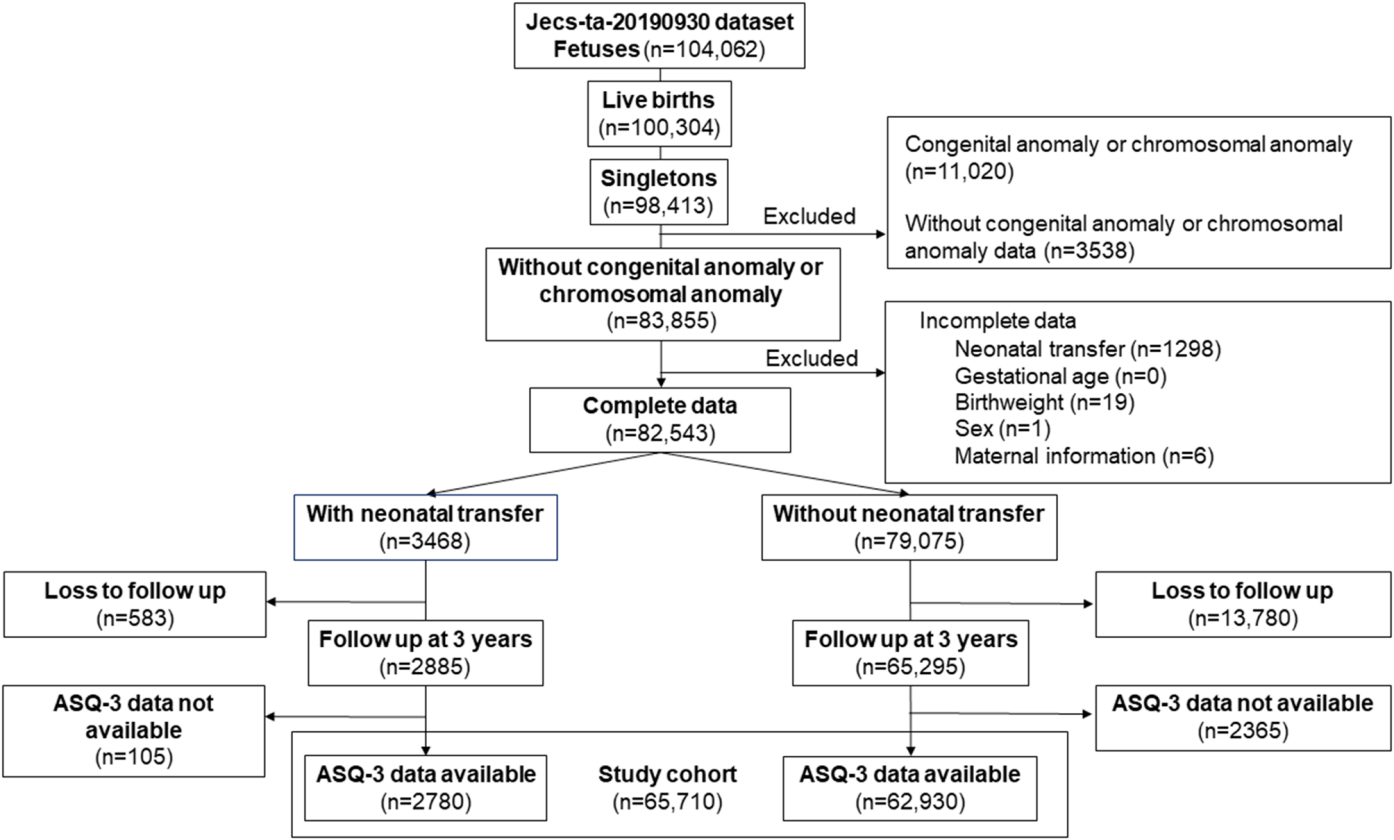
Flow diagram of patients’ enrollment in this study. ASQ-3, Ages and Stages Questionnaire, third edition

Among 104,062 fetuses in the JECS dataset, 98,413 were live-born singletons. After excluding neonates with congenital or chromosomal anomalies, or those with incomplete data of neonatal transfer, GA, birthweight, sex, or maternal information, 82,543 neonates were eligible for the study. Among them, 3468 (4.2%) were transferred to different institutes in the neonatal period, and the remaining 79,075 controls remained and were discharged from the institute where they were born. At 3 years of age, 83.2% (2885/3468) of children in the neonatal transfer group and 82.6% (65,295/79,075) in the non-neonatal transfer group were followed up. Among them, 2780 (96.4%) in the neonatal transfer group and 62,930 (96.4%) in the non-neonatal transfer group had ASQ-3 test results. Therefore, we analyzed these 65,710 children as the full study cohort (Fig. 1).

Socioeconomic background characteristics with or without neonatal transfer are shown in Table 1. In the full cohort, most of the background characteristics were similar in the 2 groups. Missing data on the mother’s educational background, household income, and drinking during pregnancy were more frequent in the neonatal transfer group compared with the non-neonatal transfer group.

### Perinatal characteristics and neonatal complications

Perinatal characteristics with and without neonatal transfer are shown in Table 2. In the full cohort, the neonatal transfer group was associated with a higher rate of primigravida, cesarean delivery, and various pregnancy-associated complications, such as HDP, GDM, placenta previa, PROM, intrauterine infection, IUGR, and NRFS. With regard to children, children who had neonatal transfer were significantly associated with a lower GA and birthweight, a higher incidence of male sex, and asphyxia at birth.

Neonatal complications in children with neonatal transfer are shown in Supplementary Fig. 1. Among 2780 transferred infants, 1799 (64.7%) were term infants and 981 (35.3%) were preterm infants. The major indications of neonatal transfer based on descriptions of neonatal complications at birth were respiratory failure (32.7%, 46.8%) and low birthweight (19.7%, 80.7%) in the term and preterm cohorts, respectively. Other indications of neonatal transfer (all < 10% in the term and preterm cohorts) were jaundice, infection, maternal complications, hypoglycemia, vomiting, asphyxia, heart murmur or arrhythmia, hypothermia, persistent pulmonary hypertension of the newborn, intraventricular hemorrhage, seizures, and unknown or others.

### Incidence of scores below the cut-off value of the ASQ-3 at 3 years of age with and without neonatal transfer

Table 3 shows the primary outcome of the study. In the full cohort, after adjustment for all potential confounders, the incidence of scores below the cut-off value of all 5 domains in the ASQ-3 was significantly higher in children with neonatal transfer compared with those without neonatal transfer (communication: odds ratio [OR] 1.42, 95% CI 1.19–1.70; gross motor: OR 1.26, 95% CI 1.07–1.49; fine motor: OR 1.19, 95% CI 1.03–1.36; problem solving: OR 1.29, 95% CI 1.12–1.48; and personal-social OR 1.52, 95% CI 1.26–1.83). Supplementary Table 1 shows alternative analysis adjusted for GA as categorical parameters instead of continuous variables.

**Table 3:** Incidence of scores below the cut-off value of the ASQ-3 at 3 years old in the full, term, and preterm cohorts with or without neonatal transfer with adjustment for perinatal confounders

	Full cohort (all GAs)				Term cohort (GA ≥ 37 weeks)				Preterm cohort (GA < 37 weeks)			
	With neonatal transfer	Without neonatal transfer	OR (95% CI)	*p* value	With neonatal transfer	Without neonatal transfer	OR (95% CI)	*p* value	With neonatal transfer	Without neonatal transfer	OR (95% CI)	*p* value
Communication (< 29.95)	179/2770 (6.5)	2201/62,719 (3.5)	1.42 (1.19–1.70)	< 0.001	89/1796 (5.0)	2125/61,054 (3.5)	1.30 (1.04–1.63)	0.02	90/974 (9.2)	76/1665 (4.6)	1.50 (1.03–2.20)	0.04
Gross motor (< 39.26)	210/2778 (7.6)	2531/62,797 (4.0)	1.26 (1.07–1.49)	< 0.001	107/1799 (5.9)	2425/61,129 (4.0)	1.30 (1.06–1.60)	0.01	103/979 (10.5)	106/1668 (6.4)	1.04 (0.74–1.48)	0.81
Fine motor (< 27.91)	312/2765 (11.3)	4449/62,566 (7.1)	1.19 (1.03–1.36)	0.01	156/1791 (8.7)	4269/60,908 (7.0)	1.10 (0.93–1.31)	0.28	156/974 (16.0)	180/1658 (10.9)	1.11 (0.84–1.46)	0.48
Problem solving (< 30.03)	298/2748 (10.8)	4259/62,222 (6.8)	1.29 (1.12–1.48)	< 0.001	149/1779 (8.4)	4118/60,566 (6.8)	1.14 (0.96–1.36)	0.13	149/969 (15.4)	141/1656 (8.5)	1.40 (1.04–1.87)	0.02
Personal-social (< 29.89)	171/2773 (6.2)	1821/62,664 (2.9)	1.52 (1.26–1.83)	< 0.001	84/1795 (4.7)	1760/60,998 (2.9)	1.39 (1.10–1.75)	0.006	87/978 (8.9)	61/1666 (3.7)	1.75 (1.17–2.62)	0.006

Data are expressed as number (%) or OR (95% CI)

Adjustment for mothers: age, marital status, primigravida, fertility treatment, cesarean delivery, epidural analgesia during labor, hypertensive disorder of pregnancy, gestational diabetes mellitus, placenta previa, premature rupture of the membranes, intrauterine infection, intrauterine growth restriction, non-reassuring fetal status, alcohol drinking during pregnancy, smoking during pregnancy, educational status, work status, and household income

Adjustment for children: gestational age, birthweight, sex, and asphyxia at birth

ASQ-3 Ages and Stages Questionnaire, third edition, *GA* gestational age, *OR* odds ratio, *CI* confidence interval

### Physician’s diagnosis of neurological impairment at 3 years of age with and without neonatal transfer

In the full cohort, after adjustment for all potential confounders, the incidence of neurodevelopmental delay (OR 1.74, 95% CI 1.15–2.62), motor developmental delay (OR 4.25, 95% CI 2.38–7.58), and cerebral palsy (OR 5.40, 95% CI 1.39–20.9) was significantly higher in 3-year-old children in the neonatal transfer group compared with that in the non-neonatal transfer group (Supplementary Table 2).

### Sensitivity analysis

Among 65,710 children in the study cohort, 63,058 (96.0%) were in the term cohort and 2652 (4.0%) were in the preterm cohort. Socioeconomic background characteristics with or without neonatal transfer in the term and preterm cohorts were not different between the 2 groups (Table 1). Additionally, similar to the full cohort, neonatal transfer in the term and preterm cohorts was associated with a higher rate of primigravida, cesarean delivery, and pregnancy-associated complications (Table 2). In children, GA and birthweight were lower, and the rate of asphyxia at birth was higher in the neonatal transfer group than in the non-neonatal transfer group (Table 2). Compared with the non-neonatal transfer group, the neonatal transfer group showed a higher incidence of ASQ-3 scores below the cut-off in the domains of communication (OR 1.30, 95% CI 1.04–1.63), gross motor (OR 1.30, 95% CI 1.06–1.60), and personal-social (OR 1.39, 95% CI 1.10–1.75) in term children, and in the domains of communication (OR 1.50, 95% CI 1.03–2.20), problem solving (OR 1.40, 95% CI 1.04–1.87), and personal-social (OR 1.75, 95% CI 1.17–2.62) in preterm children (Table 3).

Nineteen-hundred and five (68.5%) children in the neonatal transfer group and 19,430 (30.9%) children in the non-neonatal transfer group stayed hospital ≥ 7 days at birth. In the cohort restricted to prolonged hospital stay at birth, the incidence of scores below the cut-off was significantly higher in the domains of communication (OR 1.28, 95% CI 1.02–1.61), gross motor (OR 1.34, 95% CI 1.09–1.63), fine motor (OR 1.19, 95% CI 1.01–1.41), problem solving (OR 1.24, 95% CI 1.04–1.47), and personal-social (OR 1.58, 95% CI 1.26–1.98) in the neonatal transfer group compared with the non-neonatal transfer group after adjustment for GA, birthweight, and sex (Table 4).

**Table 4:** Incidence of scores below the cut-off value of the ASQ-3 at 3 years old in the cohort with prolonged hospital stay at birth (≥ 7 days) with or without neonatal transfer with adjustment for perinatal confounders

	With neonatal transfer (*n* = 1905)	Without neonatal transfer (*n* = 19,430)	Crude OR (95% CI)	*p* value	Adjusted OR (95% CI)	*p* value
Communication (< 29.95)	121/1898 (6.4)	724/19,372 (3.7)	1.75 (1.44–2.14)	< 0.001	1.28 (1.02–1.61)	0.03
Gross motor (< 39.26)	154/1904 (8.1)	900/19,398 (4.6)	1.81 (1.51–2.16)	< 0.001	1.34 (1.09–1.63)	0.005
Fine motor (< 27.91)	228/1895 (12.0)	1555/19,313 (8.1)	1.56 (1.35–1.81)	< 0.001	1.19 (1.01–1.41)	0.04
Problem solving (< 30.03)	211/1882 (11.2)	1411/19,199 (7.3)	1.59 (1.34–1.86)	< 0.001	1.24 (1.04–1.47)	0.02
Personal-social (< 29.89)	123/1900 (6.5)	654/19,357 (3.4)	1.98 (1.62–2.41)	< 0.001	1.58 (1.26–1.98)	< 0.001

Data are expressed as number (%) or OR (95% CI)

Adjustment for children: gestational age, birthweight, sex

*ASQ-3* Ages and Stages Questionnaire, third edition, *OR* odds ratio, *CI* confidence interval

One hundred thirty-two (4.7%) children in the neonatal transfer group and 141 (0.2%) children in the non-neonatal transfer group had asphyxia at birth (5-min Apgar score < 7). The incidence of scores below the cut-off was significantly higher in the domains of communication (OR 5.98, 95% CI 1.62–22.1), problem solving (OR 5.54, 95% CI 1.95–15.7), and personal-social (OR 4.59, 95% CI 1.22–17.3) in the neonatal transfer group compared with the non-neonatal transfer group after adjustment for GA, birthweight, and sex (Table 5).

**Table 5:** Incidence of scores below the cut-off value of the ASQ-3 at 3 years old in the cohort with asphyxia at birth (5-min Apgar score < 7) with or without neonatal transfer with adjustment for perinatal confounders

	With neonatal transfer (*n* = 132)	Without neonatal transfer (*n* = 141)	Crude OR (95% CI)	*p* value	Adjusted OR (95% CI)	*p* value
Communication (< 29.95)	17/132 (12.9)	3/140 (2.1)	6.75 (1.93–23.6)	0.003	5.98 (1.62–22.1)	0.007
Gross motor (< 39.26)	16/132 (12.1)	7/140 (5.0)	2.62 (1.04–6.59)	0.04	1.17 (0.40–3.45)	0.77
Fine motor (< 27.91)	20/132 (15.2)	16/139 (11.5)	1.37 (0.68–2.78)	0.38	1.13 (0.52–2.47)	0.76
Problem solving (< 30.03)	24/131 (18.3)	5/137 (3.6)	5.92 (2.19–16.0)	0.0005	5.54 (1.95–15.7)	0.001
Personal-social (< 29.89)	15/132 (11.4)	3/140 (2.1)	5.85 (1.65–20.7)	0.006	4.59 (1.22–17.3)	0.02

Data are expressed as number (%) or OR (95% CI)

Adjustment for children: gestational age, birthweight, sex

*ASQ-3* Ages and Stages Questionnaire, third edition, *OR* odds ratio, *CI* confidence interval

## Discussion

The present study is the first to investigate long-term outcomes of outborn newborns who required neonatal transfer in the general population based on a prospective cohort. We found a significant association between neonatal transfer and 3-year neurological impairment in the full, term, and preterm cohorts. Notably, these results were consistent when restricted to the children with prolonged hospital stay or asphyxia at birth. Previous reports on preterm infants have shown that outborn birth is associated with short-term adverse neurological outcomes [9–18]. However, few reports have focused on long-term outcomes of outborn preterm infants. The Canadian Neonatal Network and Canadian Neonatal Follow-up Network reported that the risks for composite outcomes of neurodevelopmental impairment and death at 18–21 months of age were higher in outborn extremely preterm infants compared with those who were inborn [18]. The Neonatal Research Network of Japan reported that the risk for cognitive impairment at 3 years of age was significantly higher in extremely preterm infants who were outborn compared with those who were inborn [17].

A higher incidence of brain injury in outborn preterm infants compared with inborn preterm infants [9–18] led to a higher risk of adverse long-term neurological outcomes [17, 18]. In the present study, after excluding congenital and chromosomal anomalies from the analysis, the major indications of neonatal transfer were respiratory failure and low birthweight in the term and preterm cohorts. Nonetheless, strikingly, our study further clarified that adverse long-term neurological outcomes in outborn infants are consistent in all populations and not exclusive to premature infants.

Two possible disadvantages in neonates with neonatal transfer are resuscitation quality in the birth hospital and medical transport in the vulnerable period. First, suboptimal resuscitation and a lack of equipment and staff expertise have been identified as risk factors contributing to adverse outcomes [24]. A retrospective study reported that outborn infants born at level I or II institutes had equivalent outcomes to inborn infants born at level IV NICUs if a neonatologist was available at deliveries [25].

Second, neonatal transfer involves additional handling, temperature instability, noise and vibration exposure, and suboptimal monitoring and ventilator management [26]. Therefore, inter-hospital transfer may induce physiological deterioration in neonates [5, 27, 28]. Higher levels of discomfort as indicated by an increase in premature infant pain profile scores were reported during transport compared with baseline [29]. Therefore, organization of a sophisticated retrieval team [30, 31] and neurocritical care of high-risk newborns during neonatal transfer [26] may improve the outcomes of outborn neonates. Whether neonatal transfer itself affects the outcome [13] or does not affect the outcome [32–34] is controversial. A few studies showed that short-term neurological outcomes of preterm infants who were transferred between tertiary-level centers were not different from non-transferred controls [10, 32]. However, these were retrospective studies, and there is a concern that neonatal transfer might induce subtle brain damage that is undetectable by cranial ultrasonography, indicating the importance of evaluating long-term outcomes.

Prenatal consultation for higher-level centers when known prenatal risks are identified could be a solution for decreasing neonatal transfer. Rates of neonatal transfer of very preterm infants differ by country and region [35]. These rates range from approximately 15 to 20% in England [36], the USA [11], and Australia [37], and 2 to 4% in Finland [35]. In previous studies, the risk of obstetric complications was lower in preterm outborn infants compared with preterm inborn infants [9–18]. This finding might be due to prenatal consultation for higher level centers when known prenatal risks are identified [9, 38]. However, in the present study of data obtained from the general population, the rate of obstetric complications was higher in the outborn population compared with the inborn population. The present study showed that the rates of neonatal transfer were 4.2%, 2.9%, and 37.0% in the full, term, and preterm cohorts, respectively. There may be room for improvement of prenatal consultations in pregnant women with obstetric complications [9–18].

The Ministry of Health, Labour and Welfare of Japan formulated guidelines that pertain to maintenance of perinatal medical systems [38]. Specifically, prefectural government bodies are required to designate tertiary and regional perinatal medical centers, which provide not only high-level intensive neonatal care, but also high-level intensive obstetric maternal–fetal care [38]. Although considerable proportions of deliveries are managed at small birth centers, Japan has achieved one of the lowest neonatal mortality rates in the world. Further establishment of sophisticated perinatal system, which involve referring high-risk pregnant women to higher-level centers and increasing delivery at perinatal centers, may reduce the incidence of neonatal transfer. These efforts could lead to improved neurological outcomes in the general population at 3 years of age [38].

There are several limitations to this study. First, we did not have detailed data on neonatal transfer, such as birth and transferred hospital volume, age at neonatal transfer, distance between centers, details on the resuscitation at birth, and during neonatal transfer data. However, in Japan, the majority of neonatal transfers are conducted between small regional birth centers/level I maternity care hospitals and level II or III perinatal centers. Previous studies have reported that the duration of neonatal transfer [39] or time from birth to neonatal transfer [40] may affect the short-term outcome. However, these detailed data are difficult to be obtained from nation-wide, large-scale prospective studies. Second, the indications of neonatal transfer were based on descriptions of neonatal complications at birth. These descriptions were based on an open-ended questionnaire answered by healthcare providers. Therefore, healthcare providers might not have documented all of the complications, which may have led to underestimating the frequency of neonatal complications. Third, the main outcome data were based on questionnaires answered by caregivers. Although the ASQ-3 was used as a screening tool for developmental delay in children, the reliability of this tool on neurodevelopment has been validated [22, 41, 42]. Fourth, we were not able to analyze the clinical severity using Transport Risk Index of Physiologic Stability scores because of insufficient data.

In conclusion, our findings provide novel evidence that neonatal transfer is associated with an increased risk of neurodevelopmental impairment as shown by scores below the cut-off value of all 5 domains in the ASQ-3 at 3 years of age.

## Electronic supplementary material

Below is the link to the electronic supplementary material.

Supplementary file1 (DOCX 62 KB)

## Data Availability

Data are unsuitable for public deposition due to ethical restrictions and legal framework of Japan. It is prohibited by the Act on the Protection of Personal Information (Act No. 57 of 30 May 2003, amendment on 9 September 2015) to publicly deposit the data containing personal information. Ethical Guidelines for Medical and Health Research Involving Human Subjects enforced by the Japan Ministry of Education, Culture, Sports, Science and Technology and the Ministry of Health, Labour and Welfare also restrict the open sharing of the epidemiologic data. All inquiries about access to data should be sent to jecs-en@nies.go.jp. The person responsible for handling enquiries sent to this e-mail address is Dr Shoji F. Nakayama, JECS Programme Office, National Institute for Environmental Studies.

## Abbreviations

ASQ-3: The Ages and Stages Questionnaire, third edition
CI: Confidence interval
GA: Gestational age
GDM: Gestational diabetes mellitus
HDP: Hypertensive disorder of pregnancy
IUGR: Intrauterine growth restriction
JECS: The Japan Environment and Children’s Study
NICUs: Neonatal intensive care units
NRFS: Non-reassuring fetal status
OR: Odds ratio
