# Correction to: Long-term outcomes of children with neonatal transfer: the Japan Environment and Children’s Study

**DOI:** 10.1007/s00431-023-04832-5

**Published:** 2023-02-01

**Authors:** Katsuya Hirata, Kimiko Ueda, Kazuko Wada, Satoyo Ikehara, Kanami Tanigawa, Tadashi Kimura, Keiichi Ozono, Hiroyasu Iso, the Japan Environment, Children’s Study Group, Michihiro Kamijima, Shin Yamazaki, Yukihiro Ohya, Reiko Kishi, Koichi Hashimoto, Chisato Mori, Shuichi Ito, Zentaro Yamagata, Hidekuni Inadera, Takeo Nakayama, Hiroyasu Iso, Masayuki Shima, Hiroshige Nakamura, Narufumi Suganuma, Koichi Kusuhara, Takahiko Katoh

**Affiliations:** 1 Department of Neonatal Medicine, Osaka Women’s and Children’s Hospital, 840 Murodo-cho, Izumi, Osaka 594-1101 Japan; 2 Osaka Maternal and Child Health Information Center, Osaka Women’s and Children’s Hospital, Izumi, Osaka Japan; 3 Public Health, Department of Social Medicine, Osaka University Graduate School of Medicine, Suita, Osaka Japan; 4 Department of Obstetrics and Gynecology, Osaka University Graduate School of Medicine, Suita, Osaka Japan; 5 Department of Pediatrics, Osaka University Graduate School of Medicine, Suita, Osaka Japan

**Correction to: European Journal of Pediatrics (2022) 181:2501-2511** 10.1007/s00431-022-04450-7

The article “Long‑term outcomes of children with neonatal transfer: the Japan Environment and Children’s Study”, written by Katsuya Hirata, Kimiko Ueda, Kazuko Wada, Satoyo Ikehara, Kanami Tanigawa, Tadashi Kimura, Keiichi Ozono, Hiroyasu Iso and the Japan Environment and Children’s Study Group, was originally published Online First without Open Access. After publication in volume 181, issue 6, page 2501–2511 the author decided to opt for Open Choice and to make the article an Open Access publication. Therefore, the copyright of the article has been changed to © The Author(s) 2023 and the article is forthwith distributed under a Creative Commons Attribution 4.0 International License, which permits use, sharing, adaptation, distribution and reproduction in any medium or format, as long as you give appropriate credit to the original author(s) and the source, provide a link to the Creative Commons licence, and indicate if changes were made. The images or other third party material in this article are included in the article's Creative Commons licence, unless indicated otherwise in a credit line to the material. If material is not included in the article's Creative Commons licence and your intended use is not permitted by statutory regulation or exceeds the permitted use, you will need to obtain permission directly from the copyright holder. To view a copy of this licence, visit http://creativecommons.org/licenses/by/4.0/.

