# Supplementary material for: Long-term outcomes of children with neonatal transfer: the Japan Environment and Children’s Study
Source: Eur J Pediatr. 2022 Mar 25;181(6):2501–11. doi: 10.1007/s00431-022-04450-7 (PMC9889501; doi:10.1007/s00431-022-04450-7)
Supplement: Supplementary file 1 — Supplementary file1 (DOCX 62 KB) [file 431_2022_4450_MOESM1_ESM.docx]

**Supplemental Information**

**Full title:** Long-term Outcomes of Children with Neonatal Transfer: the Japan Environment and Children’s Study

**Journal:** European Journal of Pediatrics

**Authors:** Katsuya Hirata, Kimiko Ueda, Kazuko Wada, Satoyo Ikehara, Kanami Tanigawa, Tadashi Kimura, Keiichi Ozono, Hiroyasu Iso; and the Japan Environment and Children’s Study Group

**Corresponding Author:**

Katsuya Hirata, MD, PhD, Department of Neonatal Medicine, Osaka Women's and Children's Hospital

Email address: khirata0513@gmail.com

**Contents:**

Supplementary Table 1. Incidence of scores below the cut-off value of the ASQ-3 at 3 years old in the full cohort with or without neonatal transfer with adjustment for perinatal confounders

Supplementary Table 2. Physician’s diagnosis of neurological impairment at 3 years old in the full cohort with or without neonatal transfer with adjustment for perinatal confounders

Supplementary Figure 1. Neonatal complications in children with neonatal transfer

**Supplementary Table 1**. Incidence of scores below the cut-off value of the ASQ-3 at 3 years old in the full cohort with or without neonatal transfer with adjustment for perinatal confounders

|  | Full cohort (all GAs) | | | |
| --- | --- | --- | --- | --- |
|  | With neonatal transfer | Without neonatal transfer | OR (95% CI) | p value |
| Communication (<29.95) | 179/2770 (6.5) | 2201/62,719 (3.5) | 1.37 (1.13-1.65) | 0.001 |
| Gross motor (<39.26) | 210/2778 (7.6) | 2531/62,797 (4.0) | 1.24 (1.04-1.27) | 0.01 |
| Fine motor (<27.91) | 312/2765 (11.3) | 4449/62,566 (7.1) | 1.13 (0.98-1.31) | 0.09 |
| Problem solving (<30.03) | 298/2748 (10.8) | 4259/62,222 (6.8) | 1.25 (1.08-1.44) | 0.003 |
| Personal-social (<29.89) | 171/2773 (6.2) | 1821/62,664 (2.9) | 1.45 (1.19-1.76) | <0.001 |

Data are expressed as number (%) or OR (95% CI). ASQ-3, Ages and Stages Questionnaire, third edition; GA, gestational age; OR, odds ratio; CI, confidence interval.

Adjustment for mothers: age, marital status, primigravida, fertility treatment, cesarean delivery, epidural analgesia during labor, hypertensive disorder of pregnancy, gestational diabetes mellitus, placenta previa, premature rupture of the membranes, intrauterine infection, intrauterine growth restriction, non-reassuring fetal status, alcohol drinking during pregnancy, smoking during pregnancy, educational status, work status, and household income.

Adjustment for children: GA, birthweight, sex, and asphyxia at birth.

We classified GA into term (≥37 weeks), late preterm (34, 35, and 36 weeks), moderate preterm (32 and 33 weeks), and very preterm (<32 weeks).

**Supplementary Table 2.** Physician’s diagnosis of neurological impairment at 3 years old in the full cohort with or without neonatal transfer with adjustment for perinatal confounders

|  | Full cohort (all GAs) | | | |
| --- | --- | --- | --- | --- |
|  | With neonatal transfer (n=2780) | Without neonatal transfer (n=62,930) | OR (95% CI) | p value |
| Neurodevelopmental impairment | 34 (1.2) | 323 (0.5) | 1.74 (1.15-2.62) | 0.008 |
| Motor developmental delay | 23 (0.8) | 76 (0.1) | 4.25 (2.38-7.58) | <0.001 |
| Cerebral palsy | 8 (0.3) | 9 (0.0) | 5.40 (1.39-20.9) | 0.01 |
| Autism spectrum disorder | 18 (0.6) | 253 (0.4) | 1.24 (0.74-2.10) | 0.42 |
| Epilepsy | 9 (0.3) | 75 (0.1) | 2.23 (0.99-5.01) | 0.05 |

Data are expressed as number (%) or OR (95% CI). GA, gestational age; OR, odds ratio; CI, confidence interval.

Adjustment for mothers: age, marital status, primigravida, fertility treatment, cesarean delivery, epidural analgesia during labor, hypertensive disorder of pregnancy, gestational diabetes mellitus, placenta previa, premature rupture of the membranes, intrauterine infection, intrauterine growth restriction, non-reassuring fetal status, alcohol drinking during pregnancy, smoking during pregnancy, educational status, work status, and household income.

Adjustment for children: gestational age, birthweight, sex, and asphyxia at birth.

**Supplementary Figure 1.** Neonatal complications in children with neonatal transfer


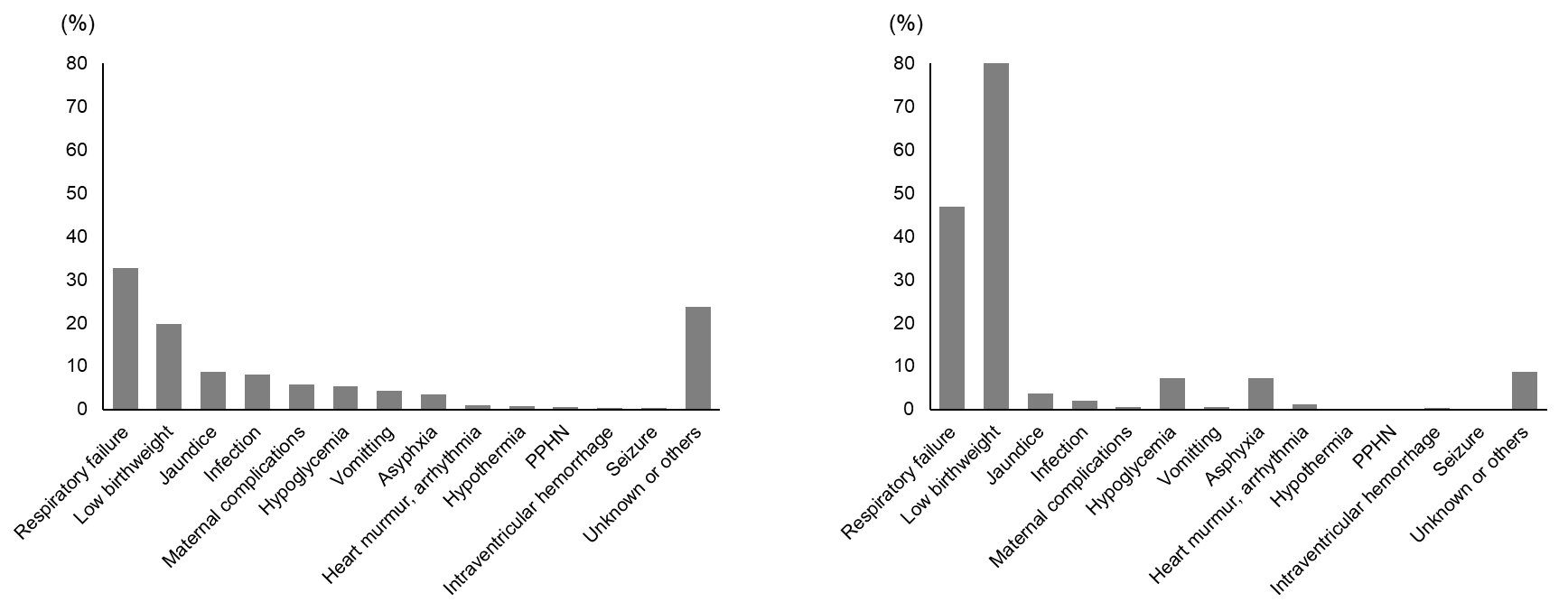


Term cohort (n=1799) Preterm cohort (n=981)

All neonates with a gestational age of ≤32 weeks were considered to have respiratory failure.

PPHN, persistent pulmonary hypertension of the newborn.
